# The Emerging JEV Genotype 5 Exhibits Distinct Codon Usage Characteristics

**DOI:** 10.3390/pathogens15010058

**Published:** 2026-01-07

**Authors:** Xiaoyu Gu, Ruichen Wang, Yuhong Yang, Weijia Zhang, Qikai Yin, Kai Nie, Shihong Fu, Qianqian Cui, Fan Li, Huanyu Wang, Songtao Xu

**Affiliations:** National Key Laboratory of Intelligent Tracking and Forecasting for Infectious Diseases, NHC Key Laboratory of Biosafety, National Institute for Viral Disease Control and Prevention, Chinese Center for Disease Control and Prevention, Beijing 102206, China; guxiaoyu905@163.com (X.G.); wangrc96@163.com (R.W.); yyh19980804@163.com (Y.Y.); zwj_0308@126.com (W.Z.); yinqk@ivdc.chinacdc.cn (Q.Y.); niekai@ivdc.chinacdc.cn (K.N.); fush@ivdc.chinacdc.cn (S.F.); cuiqq@ivdc.chinacdc.cn (Q.C.); lifan@ivdc.chinacdc.cn (F.L.)

**Keywords:** Japanese encephalitis virus, genotype 5, codon usage bias, host adaptation

## Abstract

This study investigates the codon usage characteristics of Japanese encephalitis virus (JEV) genotype 5 (G5). Based on 339 complete JEV genome sequences, we systematically compared the codon usage patterns of G5 with other genotypes (G1–G4) using a multi-faceted approach, including evolutionary analysis, nucleotide composition, Relative Synonymous Codon Usage (RSCU), Principal Component Analysis (PCA), Effective Number of Codons Plot analysis (ENC-Plot), Parity Rule 2 analysis (PR2), Neutrality plot analysis, dinucleotide abundance analysis and Codon Adaptation Index analysis (CAI). The results indicate that G5 forms a distinct evolutionary branch, with both its overall GC content (50%) and GC content at the third codon position (GC3, 53%) being lower than those of other genotypes. RSCU analysis revealed a preferential use of A/U-ended codons in G5, indicating a trend towards reduced GC3 usage. ENC analysis demonstrated a stronger codon usage bias in G5 (mean ENC = 54.2). Furthermore, ENC-plot, PR2, and neutrality plot analyses collectively suggested that G5 is subject to stronger natural selection pressure. Analysis of dinucleotide abundance showed a significant increase in CA values in G5, while CAI analysis indicated higher translational efficiency in human hosts compared to Culex mosquito hosts. Our findings suggest that G5 JEV, potentially through reduced Cytosine-phosphate-Guanine (CpG) usage and optimized codon preference, may enhance its capabilities for immune evasion and host adaptation, and could possess the potential for efficient replication in humans or other mammalian hosts. This research provides crucial theoretical insights into the molecular evolutionary mechanisms of G5 JEV and informs related vaccine development.

## 1. Introduction

Japanese encephalitis virus (JEV), a mosquito-borne flavivirus, is the causative agent of Japanese encephalitis (JE), a significant endemic encephalitis. JE typically manifests as fever, meningitis, or encephalitis, and is often accompanied by impaired consciousness, seizures, focal neurological deficits, and motor disturbances [1,2]. The case fatality rate of JE can be as high as 18%, and approximately 45% of survivors suffer from permanent neurological or psychiatric sequelae [3]. Although vaccination has significantly reduced the incidence and mortality of JE, it poses an additional burden in some countries, such as Bangladesh, India, and the Philippines [4]. Consequently, in 24 countries and regions in Southeast Asia and the Western Pacific where JEV is transmitted, over 3 billion people remain at risk of infection [5].

Japanese encephalitis virus (JEV) was first identified in 1871 during an encephalitis epidemic in Japan, with two major outbreaks subsequently recorded in 1924 and 1935. The first JEV strain was successfully isolated from brain tissue of a fatal case in 1935 [6]. Its genome consists of a single-stranded, positive-sense, non-segmented RNA approximately 11 kb in length. This genome contains a long open reading frame (ORF) that encodes a single polyprotein, which is post-translationally processed into three structural proteins (C, prM, and E) and seven non-structural proteins (NS1 through NS5). The ORF is flanked by 5′ and 3′ untranslated regions [7]. The E protein, the major antigenic structural protein, mediates receptor binding and membrane fusion and serves as the primary target for neutralizing antibodies [8]. Based on the E gene sequence, JEV is classified into five genotypes: Genotype 1 (G1), Genotype 2 (G2), Genotype 3 (G3), Genotype 4 (G4), and Genotype 5 (G5) [9]. Since the 1940s, numerous JEV strains have been isolated across various regions of China. Before 2000, G3 predominated among circulating strains, while G1 and G3 have co-circulated since then. Since 2007, G1 has re-emerged as the dominant genotype in natural transmission cycles [10]. Historically, G3 was the major causative agent of JE and the predominant genotype across much of Asia. However, studies have shown that G1 has largely replaced G3 as the most frequently isolated genotype in many Asian countries, including China, Thailand, South Korea, Japan, Malaysia, Vietnam, and India [11]. G2 is primarily distributed in northern Australia and Southeast Asia, while G4 and G5 considered more ancient and genetically divergent than the other three genotypes, are typically found in tropical Southeast Asia. G5, originally identified as the Muar strain, was first isolated in Malaysia in 1952 and is recognized as the most ancient and genetically diverse genotype [6,12,13]. In recent years, G5 JEV has re-emerged. Following its isolation in China (Tibet) in 2009 and in South Korea in 2010, G5 cases have been detected in multiple regions of South Korea since 2010 [14,15,16], indicating geographical expansion and underscoring the need for increased attention, highlighting the critical need for in-depth research focused on the G5.

Codon Usage Bias (CUB) serves as a key molecular signature of viral host adaptation and evolution, influencing viral replication efficiency, virulence, and immune evasion capabilities [17]. Studies indicate that flaviviruses generally exhibit a preference for U- and A-ended codons, with an average GC content of approximately 50.5% and a mean Effective Number of Codons (ENC) of 55.56. A significant correlation is observed between the GC content at the third codon position (GC3s) and ENC. Results from ENC analysis, neutrality plots, and correlation analyses collectively demonstrate that natural selection is the predominant force shaping their codon usage bias [18]. Consistent with this pattern across the family, JEV genotypes overall also display a codon usage bias primarily influenced by natural selection [19]. Over the past decades, G1 and G3 have been the predominant genotypes circulating globally, and their ecological, epidemiological, and molecular characteristics have been relatively well-studied [20]. In contrast, a systematic analysis of the codon usage features specific to the distinct G5 remains lacking.

Therefore, this study focuses on the genomic analysis of G5. We aim to understand the macro-level characteristics of the genome and nucleotide composition, thereby elucidating the fundamental framework of the virus. Relative Synonymous Codon Usage (RSCU) was analyzed to examine the relative probability of a specific codon being used among its synonymous codons encoding the same amino acid [21]. Principal Component Analysis (PCA) was then applied to the RSCU results as a data dimensionality reduction algorithm [22] to identify the principal components representing characteristic patterns. Effective Number of Codons (ENC) analysis was conducted primarily to assess the strength of codon usage bias [23]. Parity Rule 2 (PR2) analysis and Neutrality plot were performed to determine whether codon usage preference is influenced by natural selection or mutational pressure [24,25]. Dinucleotide abundance analysis was carried out to investigate the frequency of adjacent nucleotide pairs [26], while Codon Adaptation Index (CAI) analysis was used to evaluate the translational efficiency of codons during gene expression [27]. This study aims to systematically elucidate the genomic characteristics of G5 Japanese encephalitis virus (JEV) compared to other genotypes, assess its potential adaptive evolution, and thereby provide critical theoretical insights and data support for predicting its epidemiological trends, evaluating the cross-protective efficacy of existing vaccines, and formulating targeted prevention and control strategies.

## 2. Materials and Methods

### 2.1. Evolutionary and Nucleotide Composition Analysis

Reference sequences were retrieved from the NCBI database (https://www.ncbi.nlm.nih.gov). A total of 339 complete or nearly complete JEV genome sequences were downloaded. The selected reference sequences represent JEV strains prevalent in different regions and time periods, each with clearly documented isolation dates and locations, and collectively cover all five genotypes, sequence information is provided in the Supplementary Table S1. A phylogenetic tree based on the Neighbor-Joining (NJ) method was constructed using MEGA 7, with bootstrap analysis performed with 1000 replicates, and the resulting tree was visualized and refined using the ChiPlot online tool “https://www.chiplot.online/normalTree.html (16 December 2025)”. Nucleotide composition analysis was conducted on the open reading frame (ORF) of all sequences. The following parameters were analyzed using DAMBE Software Version 7.0.35 [28]: the content of individual bases (A, U, C, G), overall GC content, overall AU content; AU or GC content at the first, second, and third codon positions (AU1, AU2, AU3, GC1, GC2, GC3). The GC12 value (average of GC1 and GC2) and AU12 value (average of AU1 and AU2) were calculated from the GC1/GC2 and AU1/AU2 contents, respectively. The analysis also included the content of individual bases at the first (A1, U1, C1, G1), second (A2, U2, C2, G2), and third (A3, U3, C3, G3) codon positions. All analyses excluded stop codons (UAA, UAG, UGA) from the calculations.

### 2.2. RSCU Analysis

Relative Synonymous Codon Usage (RSCU) represents the relative probability of a specific codon being used among synonymous codons encoding the same amino acid. RSCU analysis was performed using the RSCUcaller package in R Software Version 2023.12.1.0 [29]. An RSCU value less than 0.6 indicates low usage frequency of the codon, while a value equal to 1 suggests no usage bias. An RSCU value greater than 1 reflects higher usage bias, whereas a value below 1 indicates lower bias, codons with RSCU values > 1.6 are defined as “over-represented”, and those with values < 0.6 as “under-represented” [21]. For each gene, the codon with the highest RSCU value and the highest occurrence frequency among synonymous codons was identified as the optimal codon [30]. The RSCU values for the ORF of different JEV genotypes were calculated, and the types and numbers of over- and under-represented codons in each genotype were statistically analyzed.

### 2.3. PCA Based on RSCU

Principal Component Analysis (PCA) is a widely used algorithm for dimensionality reduction [22]. In this study, PCA was performed on the RSCU values by computing the covariance matrix, followed by eigenvalue decomposition. Principal components were selected based on the magnitude of the eigenvalues. The analysis was conducted using the prcomp() function in R.

### 2.4. ENC-GC3s Plot Analysis

The Effective Number of Codons (ENC) is a key metric for evaluating codon usage bias. The ENC value ranges from 20 to 61, with a cutoff value of 35 commonly used to assess the extent of bias. An ENC value greater than 35 indicates relatively low codon usage bias, while a value below 35 suggests higher bias [23].

The ENC-GC3s plot analysis examines the relationship between GC3 content and ENC values, thereby reflecting the influence of GC3 on codon usage bias. The expected ENC value for each GC3 content was calculated and used to plot the standard ENC curve according to the formula (Formula (1)). A scatter plot was then generated with the GC3 content of each JEV sequence as the *x*-axis and its corresponding ENC value as the *y*-axis. When data points are distributed on or near the standard curve, it indicates that codon usage bias is influenced solely by mutation pressure. Conversely, when data points fall below the standard curve, it suggests the influence of natural selection pressure [31].

(1) $${ENC}_{expected}=2+s+29/\left[s^{2}+{\left(1-s\right)}^{2}\right]$$

This expression describes the standard ENC curve, where *s* denotes the GC content at the third codon position, and *ENC_expect* represents the expected ENC value for the corresponding GC3 content.

### 2.5. Parity Rule 2 (PR2) Analysis

Parity Rule 2 (PR2) analysis, also referred to as parity rule preference analysis, was employed to assess base composition bias. A scatter plot was constructed with A3/(A3 + U3) as the *x*-axis and G3/(G3 + C3) as the *y*-axis for each JEV sequence. When data points cluster around the central point (0.5, 0.5), it indicates parity between A3 and U3, as well as between C3 and G3, suggesting that mutation pressure is the sole factor influencing viral codon usage bias. Conversely, significant deviation of data points from the center indicates substantial disparities in the usage frequency of the four bases, demonstrating that codon usage bias is also shaped by natural selection pressure [24].

### 2.6. Neutrality Plot Analysis

The neutrality plot was used to evaluate the relative contributions of mutation pressure and natural selection to genomic evolution. A scatter plot was generated with GC3 as the *x*-axis and GC12 as the *y*-axis, and a regression line was fitted for neutrality analysis. The regression coefficient reflects the influence of mutation pressure versus natural selection on codon usage bias. A regression coefficient close to 1 suggests that the base composition among codon positions is highly similar, indicating that codon usage bias is predominantly governed by mutation pressure. In contrast, a regression coefficient deviating substantially from 1 implies a greater role for natural selection pressure in shaping codon usage bias [25].

### 2.7. Dinucleotide Abundance Analysis

Dinucleotide abundance is a metric used to quantify whether the observed frequency of any two adjacent nucleotides in a sequence deviates from the theoretical expectation. Dinucleotide abundance values were calculated using DAMBE software “http://dambe.bio.uottawa.ca/DAMBE/dambe.aspx (23 September 2025)”. The abundance value for each dinucleotide was computed using the following formula (Formula (2)):

(2) $$P=\frac{fxy}{fxfy}$$

where *fx* and *fy* represent the respective frequencies of nucleotides *x* and *y* in the sequence, and *fxy* denotes the frequency of the dinucleotide *xy*. A *Pxy* value significantly greater than 1.23 indicates over-representation, suggesting higher-than-expected abundance of the dinucleotide; conversely, a value less than 0.78 signifies under-representation, reflecting a deficiency in its abundance [26].

### 2.8. Codon Adaptation Index (CAI) Analysis

The Codon Adaptation Index (CAI) is a widely used metric for predicting gene expression levels, particularly translation efficiency. It defines codons that occur with high frequency in highly expressed genes as translationally optimal codons [27]. A CAI value approaching 1 indicates that the codon usage pattern of a gene closely matches that of the host’s highly expressed genes, suggesting a potentially high expression level. Conversely, a CAI value near 0 reflects poor concordance and predicts low expression efficiency.

## 3. Results

### 3.1. Phylogenetic and Nucleotide Composition Analysis of JEV Genotypes

**Figure 1:**
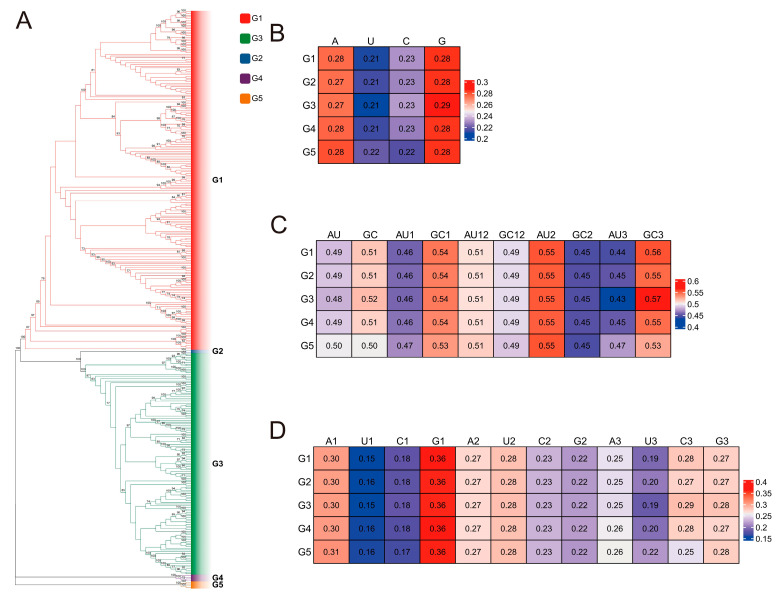
Base Analysis of JEV genome. (**A**). Evolution analysis tree of JEV. (**B**). Total base composition of JEV genome of different genotypes. (**C**). Utilization frequency of AU or CG. (**D**). Base composition of different bits of codon of different genotypes of JEV. Branch node values represent bootstrap support values based on 1000 replications, with values below 70% hidden.

Phylogenetic analysis reveals that the G5 of Japanese encephalitis virus (JEV) occupies a relatively independent branch, indicating a more distant genetic relationship with the other four genotypes (G1–G4). Comparative analysis of overall nucleotide composition showed distinct patterns among the genotypes. The total base composition for G1 and G4 was G = A > C > U, while for G2 and G3 it was G > A> C > U. In contrast, the G5 genotype exhibited a unique profile of G = A > C = U. The overall GC content in genotypes G1–G4 was slightly higher than their AU content. Notably, the G5 displayed an equal proportion of GC and AU (50% each), with its total GC content being marginally lower (approx. 51% in others) and its AU content marginally higher than the other genotypes. Analysis of nucleotide composition across codon positions revealed further distinctions. In genotypes G1–G4, the AU and GC content across positions followed the patterns AU2 > AU1 > AU3 and GC1 > GC3 > GC2, respectively. The G5, however, displayed a different pattern: AU2 > AU1 = AU3 and GC1 = GC3 > GC2. All five genotypes shared a common feature of AU12 > GC12. At the first codon position, the base composition was consistent across all genotypes: G1 > A1 > C1 > U1. The second position was also conserved, with a composition of U2 > A2 > C2 > G2 for all genotypes. The most significant divergence was observed at the third codon position. G1, G3, and G4 shared the pattern C3 > G3 > A3 > U3, while G2 showed C3 = G3 > A3 > U3. Strikingly, the G5 genotype demonstrated a markedly different third-position composition: G3 > A3 > C3 > U3 (Figure 1).

### 3.2. Relative Synonymous Codon Usage (RSCU) Analysis

**Figure 2:**
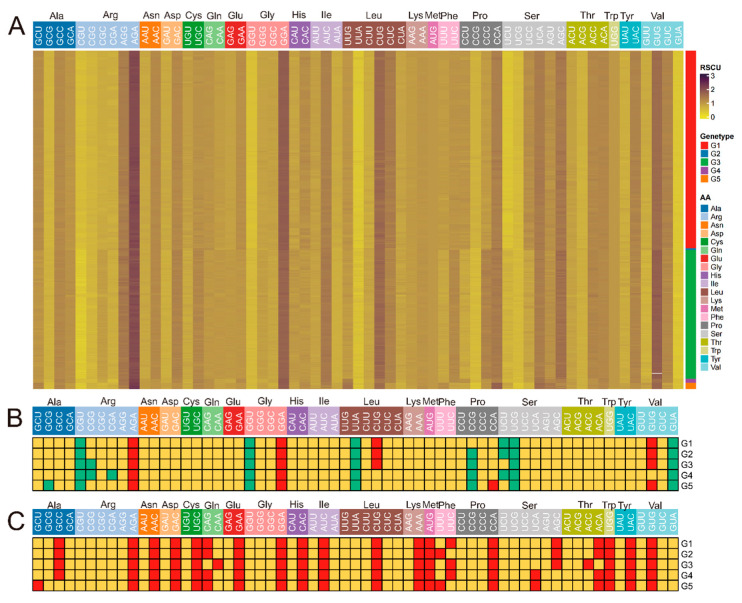
RSCU analysis. (**A**). Heat map of RSCU value of JEV. (**B**). Map of underuse, normal use and overuse codon of each genotype. (**C**). Map of optimal codon use by genotype. In panel (**B**), red represents over-represented codons, yellow represents normally used codons, and green indicates under-represented codons; in panel (**C**), red denotes optimally used codons, while yellow indicates codons with normal usage.

Analysis of relative synonymous codon usage revealed subtle differences in codon selection among the five JEV genotypes during amino acid encoding. A consistent pattern observed across all genotypes was a predominant preference for U3-ended codons, followed by A3-ended codons. Furthermore, all genotypes shared common over-represented codons for arginine (AGA) and glycine (GGA), as well as common under-represented codons for arginine (CGU), glycine (GGU), leucine (UUA), serine (UCG), and valine (GUA). Notably, genotype G5 exhibited distinct codon preferences that suggest a trend toward reduced GC3 usage. Specifically, G5 showed the strongest preference for GCU over GCC for alanine encoding, UUU over UUG for phenylalanine encoding, and UCA over AGC for serine encoding. This observed pattern in G5 is consistent with its lower overall GC content compared to other genotypes, as identified in the nucleotide composition analysis (Figure 2).

### 3.3. Principal Component Analysis Based on RSCU

**Figure 3:**
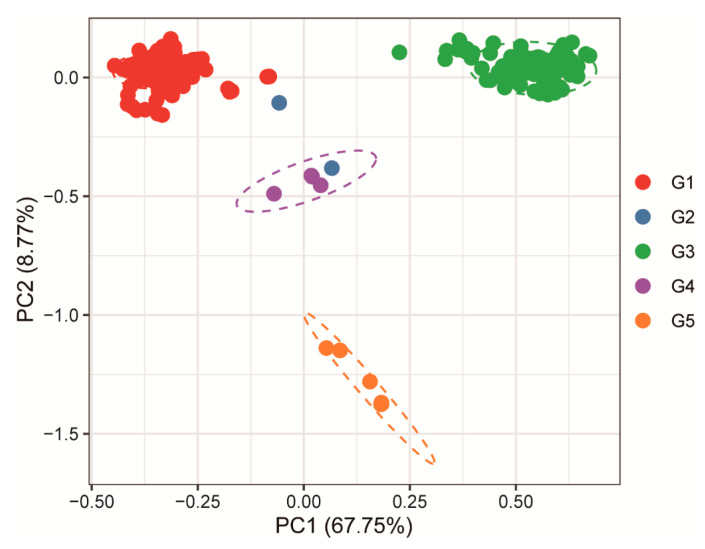
Principal component analysis (PCA) of codons of different genotypes of JEV usage variation. The horizontal axis (PC1) represents the first principal component, explaining 67.75% of the total variance in the data, while the vertical axis (PC2) corresponds to the second principal component, accounting for 8.77% of the variance. The circles of different colors in the figure represent the 95% confidence intervals of distinct genotypes, with the blue circle indicating that the sample size of the G2 genotype was insufficient for reliable clustering.

Based on the principal component analysis (PCA) of relative synonymous codon usage (RSCU), principal component 1 (PC1) accounted for 67.75% of the total variation and served as the primary factor distinguishing the JEV genotypes, whereas PC2 explained only 8.77%. This result indicates significant differences in codon usage patterns among the genotypes and reveals clear clustering characteristics. With the exception ofG2, which could not form a distinct cluster due to limited sample size, G1 and G3 were located close to each other along the PC1 axis, suggesting similar codon usage preferences. In contrast, G4 and G5 were clearly separated from the other groups, showing greater divergence especially along the PC2 axis. Moreover, the 95% confidence ellipses of the four genotypes showed no overlap, further supporting the existence of distinct codon usage patterns among JEV genotypes, which may reflect their unique host adaptation strategies or evolutionary pathways. Overall, these findings demonstrate that JEV genotypes have evolved significant codon usage biases during evolution, and that G4 and G5 in particular may have adapted to different biological environments through unique translational regulation mechanisms (Figure 3).

### 3.4. ENC-Plot Analysis

**Figure 4:**
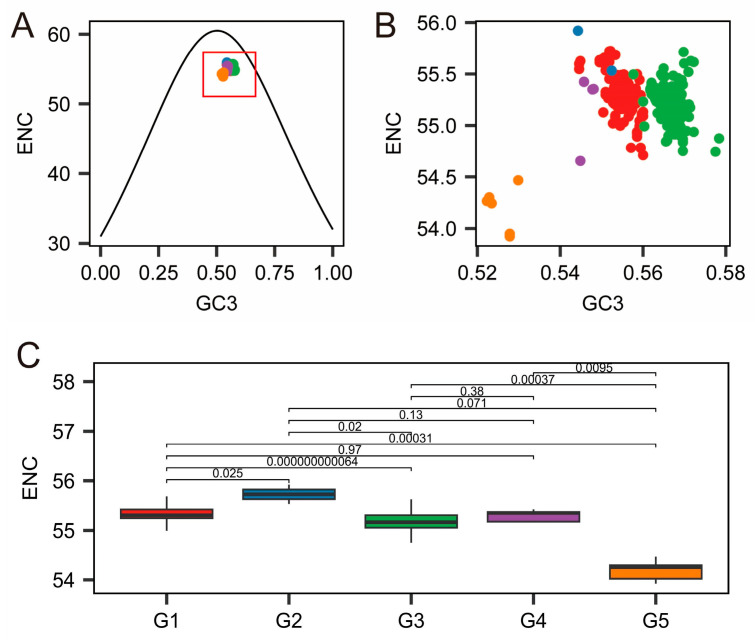
ENC analysis. (**A**). ENC-CG3 analysis (**B**). The red box in A is enlarged. (**C**). Box pattern of ENC of each genotype. The *X*-axis (GC3) represents the GC content at the third codon position, scaled from 0 to 1. The *Y*-axis (ENC) indicates the effective number of codons. A lower ENC value corresponds to stronger codon usage bias, while a higher ENC value reflects weaker bias. Red, blue, green, purple, and orange dots represent genotypes G1, G2, G3, G4, and G5, respectively.

The ENC plot revealed evolutionary constraints among the five JEV genotypes from the perspective of codon usage preference. The ENC-GC3 relationship showed that most data points closely followed or approached the standard curve, indicating that JEV codon usage bias is primarily driven by mutation pressure. However, some points located below the curve suggest that natural selection also plays a role. Box plot analysis further quantified the preference intensity across genotypes: G3 exhibited the lowest ENC value, indicating the strongest bias; G1, G2, and G4 showed intermediate levels; while G5 demonstrated the greatest deviation from the standard curve, suggesting that natural selection pressure has a more pronounced influence on this genotype compared to others. These findings indicate that JEV has developed varying degrees of translational optimization during evolution, with G3 potentially undergoing the strongest natural selection for efficient translation adaptation, while G5 maintains relatively higher codon usage flexibility (Figure 4).

### 3.5. Parity Rule 2 (PR2) Analysis

**Figure 5:**
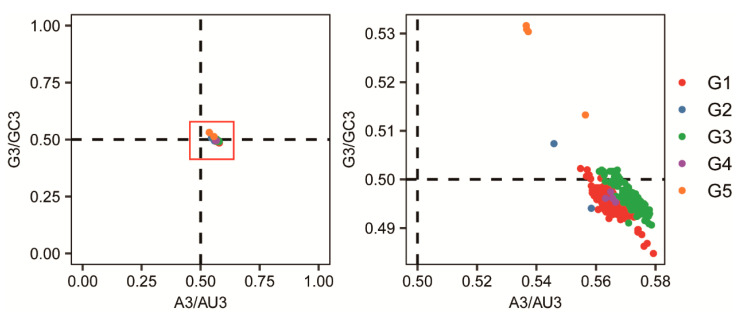
Parity Rule 2 analysis. The *X*-axis (A3/AU3) represents the ratio of adenine (A) content to the total adenine plus uracil (A + U) content at the third codon position. The *Y*-axis (G3/GC3) indicates the ratio of guanine (G) content to the total guanine plus cytosine (G + C) content at the third codon position.

In the PR2 analysis, the data points representing different JEV isolates were all distributed distantly and non-uniformly from the central point (0.5, 0.5), indicating imbalanced usage of bases at the third codon position and demonstrating that JEV codon usage is influenced by both mutation pressure and natural selection. All data points were located to the right of the vertical line X = 0.5, revealing that A3 > U3 across JEV genotypes. The data points corresponding to G1, G3, and G5 were situated below the horizontal line Y = 0.5, indicating G3 < C3 in these three genotypes. In contrast, the data points for G5 were positioned above Y = 0.5, demonstrating that G3 > C3 in this specific genotype (Figure 5).

### 3.6. Neutrality Plot Analysis

**Figure 6:**
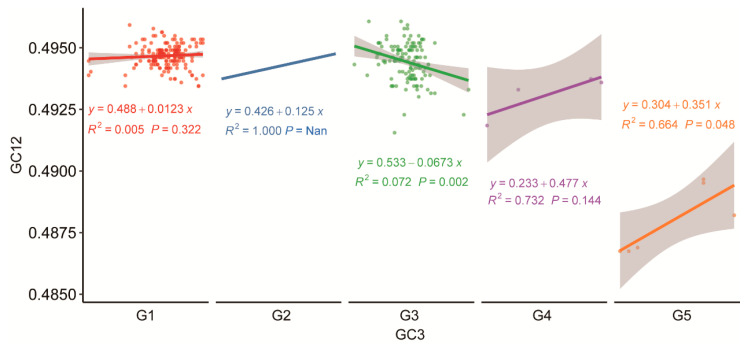
Neutrality picture analysis. The *X*-axis (GC3) indicates the GC content at the third codon position across different JEV genotypes, which is typically most influenced by mutational pressure. The *Y*-axis (GC12) represents the GC content at the first and second codon positions, which are generally more susceptible to the effects of natural selection.

In the neutrality plot analysis, the absolute value of the regression slope indicates the degree of influence exerted by mutation pressure. The calculated absolute slope values for JEV G1 to G5 were 0.0123, 0.125, 0.0673, 0.477, and 0.351, respectively. These values indicate that the relative contribution of mutation pressure to codon usage bias was 1.23%, 12.5%, 6.73%, 47.7%, and 35.1% for genotypes G1 to G5, respectively. Consequently, the corresponding influence of natural selection pressure, calculated as 1 minus the contribution of mutation pressure, was determined to be 98.77%, 87.5%, 93.27%, 52.3%, and 64.9% for G1 to G5, respectively. These findings are consistent with the results from the ENC-plot analysis, confirming that natural selection represents the dominant evolutionary force shaping codon usage patterns across JEV genotypes. Neutrality plot analysis indicates that genotypes G4 and G5 are subject to stronger mutational pressure compared to the other three genotypes (Figure 6).

### 3.7. Dinucleotide Abundance Analysis

**Figure 7:**
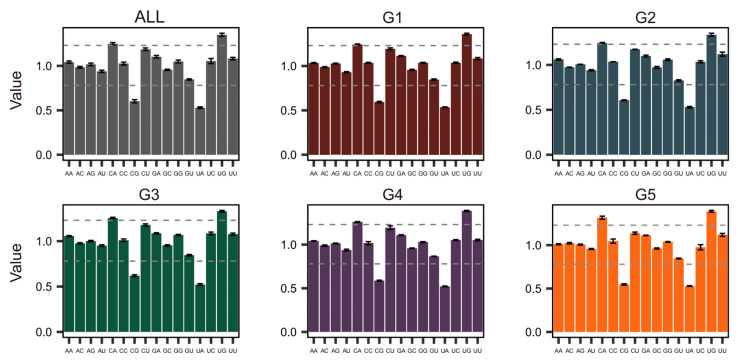
Dinucleotide abundance value of JEV genome The *X*-axis denotes dinucleotides, while the *Y*-axis represents dinucleotide abundance values, with higher values indicating higher frequencies of dinucleotide usage and lower values indicating lower frequencies. The dashed interval represents moderate dinucleotide usage frequency.

Analysis of dinucleotide abundance revealed distinct patterns in dinucleotide usage frequency across the JEV genome. Deviation from expected dinucleotide abundance values reflects the combined effects of mutation pressure and natural selection during viral evolution. Building upon previous findings of both shared and divergent codon usage patterns among genotypes, we inferred that different genotypes might exhibit distinct dinucleotide usage profiles. The dinucleotide abundance patterns were generally consistent across genotypes and with the overall abundance profile. Specifically, CG and UA consistently showed the lowest abundance values, while UG displayed the highest abundance, followed by CA and CU. However, G5 exhibited a marked increase in CA dinucleotide abundance compared to the other four genotypes and the overall average (Figure 7).

### 3.8. Codon Adaptation Index (CAI) Analysis

**Figure 8:**
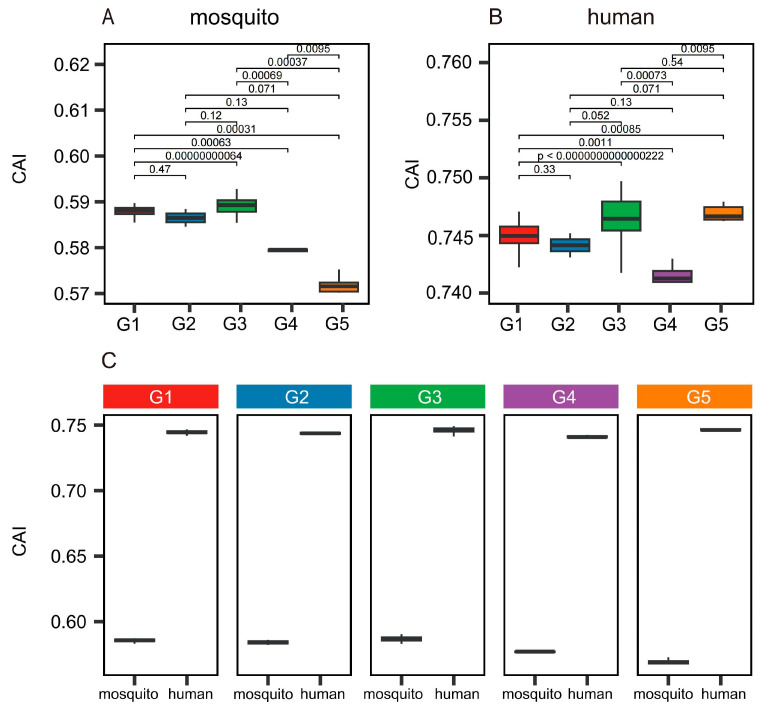
CAI analysis of JEV genome (**A**): CAI analysis of the JEV genome in Culex; (**B**): CAI analysis of the JEV genome in humans; (**C**): comparative CAI analysis of JEV in human versus Culex hosts. In panels (**A**,**B**), the *X*-axis represents different JEV genotypes, and the *Y*-axis indicates the CAI values. In panel (**C**), the *X*-axis displays JEV genotypes G1 through G5 from left to right, with the left side of each genotype corresponding to mosquitoes and the right side to humans; the *Y*-axis similarly denotes CAI values.

Codon Adaptation Index (CAI) analysis revealed differences in codon translation efficiency among the five JEV genotypes across different host species. In the Culex mosquito host, all genotypes exhibited relatively high CAI values with limited variation (ranging from 0.57 to 0.62). Among these, G3, G1, and G2 demonstrated the highest anticipated expression levels, indicating superior codon translation efficiency and consequently higher protein synthesis capacity in mosquitoes. The G5 type exhibits the lowest level, indicating that its expected expression in Culex mosquitoes is low, with reduced codon translation efficiency leading to minimal protein synthesis. In human hosts, the CAI values are predominantly concentrated within the 0.74–0.76 range. Although the CAI values for all genotypes are higher than those observed in Culex mosquitoes, the overall trend remains largely consistent. However, the CAI value of the G5 stands out as a notable exception, showing a significant increase in human hosts. This indicates that the codon translation efficiency of G5 is markedly higher in human hosts compared to that in Culex mosquito hosts (Figure 8).

## 4. Discussion

This study systematically deciphers the codon usage patterns of G5 and compares its sequence characteristics with other JEV genotypes, revealing unique evolutionary traits specific to G5. A total of 339 sequences were downloaded from NCBI, but some sequence information was incomplete. Additionally, the number of sequences obtained for each of the five genotypes varied, which introduced a potential bias into the sequence analysis. Genotypes with more sequences (G1 and G3) yielded relatively representative analytical outcomes, whereas those with fewer sequences (G2, G4, and G5) produced results that should be interpreted with greater caution. Therefore, while the findings of this study provide useful insights into the mechanisms of host adaptation and the molecular basis of enhanced pathogenicity, a more comprehensive understanding of the characteristic differences between G5 and the other four genotypes would require the inclusion of a larger number of sequences in the analysis. Although G5 shares general codon usage preferences with other genotypes, its specific patterns exhibit distinct characteristics. Phylogenetic analysis suggests that G5 may have developed unique adaptive strategies during its evolution. Analysis of nucleotide composition revealed that G5 displays a distinctive profile: equal proportions of G and A, as well as equal proportions of C and U, resulting in a total GC content of 50% slightly lower than G1–G4 and correspondingly higher AU content. Positional nucleotide analysis demonstrated that G5 exhibits equal AU1 and AU3 content, as well as equal GC1 and GC3 content. Notably, the third position base composition is dominated by G3, in sharp contrast to other genotypes where C3 predominates. RSCU analysis indicated that G5 exhibits unique preference patterns for specific amino acids. For alanine, G5 shows strongest preference for GCU rather than the GCC commonly used by other genotypes. For phenylalanine, it favors UUU over UUG, and for serine, it prefers UCA rather than AGC. Collectively, these preferences reflect G5′s tendency to reduce G and C usage at the third codon position, consistent with its lower overall GC content. PCA based on RSCU values revealed that PC1 accounts for 67.75% of the variation, serving as the key factor differentiating genotypes, while PC2 explains only 8.77%. Although G2 did not form a distinct cluster due to limited sequences, G1 and G3 positioned closely along the PC1 axis, indicating similar codon usage patterns between them. In contrast, G4 and G5 clearly separated from other groups, showing greater differentiation particularly along the PC2 axis. The 95% confidence ellipses of the four genotype groups showed no overlap. Codon preference scatter plot analysis demonstrated that G5 exhibits A3 > U3 and G3 > C3 characteristics in third position base usage, contrasting sharply with the G3 < C3 pattern observed in other genotypes. Dinucleotide abundance analysis revealed that G5 has clearly higher CA dinucleotide usage compared to other genotypes, although all genotypes generally showed low CG and UA usage, and high UG and CA usage.

Natural selection pressure serves as the predominant evolutionary force shaping codon usage patterns in Japanese encephalitis virus (JEV). The ENC analysis revealed that the data points of genotype 5 (G5) were located farthest from the standard ENC-GC3 curve. In cross-genotype comparisons, the ENC values of G5 were largely similar to those of other genotypes, indicating that the strength of codon usage bias is comparable across all five genotypes. Neutrality plot analysis further quantified that mutation pressure contributes 35.1% to the codon usage variation in G5, a substantially higher proportion than observed in G1, G2, and G3 genotypes, though slightly lower than that observed in G4. This confirms that the evolutionary trajectory of G5 is predominantly driven by natural selection, with mutation pressure playing a secondary role, with relatively less influence from mutational pressure, albeit to a slightly greater extent than that observed in genotypes G1, G2, and G3. The host adaptation pattern of G5 displays distinct characteristics compared to other genotypes. In the Culex mosquito host, G5 exhibited the lowest Codon Adaptation Index (CAI) value among all genotypes, indicating poor matching between its codon usage pattern and the preferred codon repertoire of highly expressed mosquito genes. This suggests that the codons of genotype 5 (G5) exhibit lower translational efficiency in Culex mosquitoes, leading to reduced protein synthesis. This may also contribute, in part, to the lower frequency with which this genotype has been associated with clinical disease. When humans serve as hosts, although the CAI values of all genotypes increase, the overall trend remains largely consistent with that observed in Culex mosquitoes. Notably, the CAI value of G5 in human hosts is significantly higher than that in mosquito hosts. This indicates that G5 possesses a slightly enhanced capacity for expression/replication in human cells compared to its performance in mosquito hosts. Comparative analysis between G5 and the other four genotypes revealed distinct nucleotide composition in G5, suggesting that its codon usage may be shaped by different regimes of natural selection and/or mutation pressure. Comparative studies with other viruses indicate that the codon usage pattern of JEV G5 differs from that of flaviviruses such as Dengue virus (DENV). While DENV exhibits a stronger preference for A/U-ended codons, primarily governed by natural selection rather than mutation pressure [32]. In contrast, JEV G5 exhibits a GC3 bias similar to that observed in West Nile virus (WNV) [33]. Compared to Venezuelan Equine Encephalitis virus (VEEV), whose codon usage closely mirrors that of its human, hamster, and equine hosts, JEV G5 appears to employ a distinct codon adaptation strategy to optimize its transmission efficiency in specific host environments [34]. RSCU analysis identified unique preferences in G5 when encoding specific amino acids, further supporting the hypothesis that G5 may adapt to host environments or optimize translational efficiency through fine-tuning its GC3 usage preference [35]. Principal component analysis (PCA) confirmed obvious differences in codon usage among JEV genotypes, revealing clear clustering patterns that underscore the distinct codon usage model of each genotype. This divergence highlights the uniqueness of G5, which may be linked to its adaptation to different hosts or transmission cycles. Notably, in WNV, a related flavivirus, codon usage in the initial coding region differs clearly from that in the overall genome, with certain codons being overrepresented in the start region despite having low RSCU in the full-length sequence [36]. Dinucleotide abundance analysis suggests that G5 may possess unique genomic structural features or RNA secondary structures, which could influence viral replication, packaging, or immune evasion mechanisms [37]. This is consistent with observations in Crimean-Congo Hemorrhagic Fever virus (CCHFV), where underrepresentation of CpG and UpA dinucleotides is thought to facilitate host immune evasion a potential parallel to the mechanism implied by the JEV G5 results [38]. In conclusion, JEV genotype G5 exhibits distinctive characteristics in nucleotide composition, codon usage, and dinucleotide preferences. These molecular differences collectively reflect a unique evolutionary trajectory for G5 and suggest specialized host interaction strategies shaped by its distinct adaptive path.

The results of ENC analysis show that the ENC value of G5 is very close to those of the other four genotypes. This indicates that all JEV genotypes exhibit a similar strength of codon usage bias and that the translational efficiency of G5 is largely consistent with that of the other genotypes. The concurrent contribution of mutation pressure (35.1%) likely directly contributed to the previously observed lower GC content relative to the other four genotypes and the systematic preference for AU ended codons in G5. This pattern is consistent with observations in SARS-CoV-2, where natural selection also predominates in shaping codon usage [39]. The lowest CAI value observed in G5 suggests potentially lower viral protein translational efficiency and replication levels in mosquito hosts, which may partly explain its relatively lower frequency of associated disease cases. The CAI value of G5 is higher in human hosts, suggesting that viral protein translational efficiency and replication levels may be greater in humans than in Culex mosquito hosts [40]. A similar host-dependent adaptation has been reported for Crimean-Congo hemorrhagic fever virus (CCHFV), which exhibits distinct codon usage patterns between human and tick hosts, with stronger adaptation observed in humans [38]. Studies on poliovirus have also indicated that a relatively lower codon usage bias may facilitate efficient replication in vertebrate host cells [41]. Furthermore, research on influenza viruses demonstrates that codon usage preferences in different subtypes can adaptively shift in response to changes in host environment during cross-species transmission [42]. The expected expression level of G5 in human hosts is higher than that of other genotypes in Culex mosquitoes, implying that it may possess the potential for more efficient replication in humans or other mammalian hosts.

## 5. Conclusions

Comparative genomic analysis between G5 and other JEV genotypes revealed distinct evolutionary characteristics in G5. Regarding nucleotide composition, G5 exhibited lower overall GC content (50%) and GC3 content (53%) compared to other genotypes (51% and 55–57%, respectively). RSCU analysis further supported the reduced usage of GC-ending codons in G5. The mean ENC value forG5 was 54.2, lower than the 55.3 observed in other genotypes, indicating a similar overall strength of codon usage bias across genotypes. PR2 analysis indicated that genotype G5 has been subjected to stronger natural selection pressure. Dinucleotide abundance profiles distinguished G5 from the other four genotypes, showing increased CA and decreased CU abundance. CAI analysis shows that all genotypes exhibit highly similar CAI values in humans, indicating comparable expression and replication capacities in human cells. However, G5 demonstrates a higher CAI value in human hosts compared to Culex mosquito hosts, suggesting the possibility of differential expression and replication ability across host species. Based on the differential analysis, it can be inferred that G5 JEV is subjected to stronger natural selection pressure, resulting in lower CpG content compared to other genotypes. This may render G5 more prone to immune evasion and more efficient at utilizing host cellular resources for replication. Consequently, G5 JEV likely possesses enhanced host adaptability.

## Data Availability

All viral genome sequences used in this study were retrieved from the National Center for Biotechnology Information (NCBI) database.

## Supplementary Materials

The following supporting information can be downloaded at https://www.mdpi.com/article/10.3390/pathogens15010058/s1. Table S1: Sequence Information Table.
